# Research Progress in and Defect Improvement Measures for Laser Cladding

**DOI:** 10.3390/ma18133206

**Published:** 2025-07-07

**Authors:** Bo Cui, Peiqing Zhou, You Lv

**Affiliations:** 1 College of Mechanical and Civil Engineering, Jilin Agricultural Science and Technology College, Jilin 132101, China; 2 College of Mechanical and Electrical Engineering, Jilin Institute of Chemical Technology, Jilin 132022, China; 19855326909@163.com

**Keywords:** material systems, process parameters, cracking, residual stresses, mitigation and prevention

## Abstract

Laser cladding, a cutting-edge surface modification technique for metals, offers a novel approach to enhancing the wear and corrosion resistance of substrates due to its rapid heating and cooling capabilities, precise control over coating thickness and dilution rates, and non-contact processing characteristics. However, disparities in the physical properties between the coating material and the substrate, coupled with the improper utilization of process parameters, can lead to coating defects, thereby compromising the quality of the coating. This paper examines the effects of material systems and process parameters on laser cladding composite coatings and shows that cracking is mainly caused by thermal and residual stresses. This article summarizes the methods for crack improvement and prevention in five aspects: the selection of processes in the preparation stage, the application of auxiliary fields in the cladding process, heat treatment technology, the use of auxiliary software, and the search for new processes and new structural materials. Finally, the future development trends of laser cladding technology are presented.

## 1. Introduction

With the advancement of industry, critical components are extensively used in various sectors, including aerospace, automotive manufacturing, and agricultural machinery. These essential parts, such as turbine blade tips, automotive brake disks, and the surfaces of large ring gears, are exposed to harsh operating conditions, including elevated temperatures, high pressures, wear, and corrosion. Consequently, there is a critical need to enhance the reliability and durability of these components. Currently, improving the wear resistance of critical mechanical components primarily involves two approaches. The first method focuses on enhancing the wear resistance of the material itself. This is achieved by replacing traditional materials with those exhibiting superior wear resistance, thereby fundamentally extending the service life of key mechanical parts [1]. However, this method often comes with higher costs. An alternative method is to apply surface treatment techniques to create wear-resistant coatings on component surfaces. At present, the common surface strengthening techniques include plasma spraying [2,3], surfacing welding [4,5], electroplating [6], cladding, etc. While plasma spraying can improve the wear resistance of the coating, it may result in interface gaps during the manufacturing process, so the coating performance cannot be significantly improved. Surfacing welding involves a narrow material selection range and makes it challenging to add WC, TiC, and other hard particle phases to the welding material so that the wear resistance of the parts cannot be greatly improved. Electroplating coating usually produces tiny cracks; the combination of coating and substrate is a physical combination, and the binding force is poor. In addition, the plating production process will produce chemical substances that cause great harm to the environment. Compared with the previous several traditional repair technologies, laser cladding is an advanced metal surface manufacturing technology that utilizes a laser as the heat source to form a composite coating on the surface of a metal substrate, offering enhanced abrasion resistance, corrosion resistance, wettability, and oxidation resistance [7]. Laser cladding offers the advantages of an extremely rapid heating and cooling speed, precise control over the coating thickness and dilution rate, and non-contact processing, which provides a new method for surface strengthening and the repair of parts.

In recent years, scholars have conducted extensive research on cladding materials and processes, and they have achieved remarkable results in preventing and improving cracks. Li et al. [8] reviewed the types and formation mechanisms of cracks and pointed out that the differences in thermal expansion between materials and the uneven distribution of microstructure in the molten pool led to excessive residual stress and the formation of cracks. Therefore, the main measures to improve cracks include the optimization of process parameters, the use of rare earth elements and trapezoidal transition layers, the preheating of the matrix, the application of auxiliary fields, etc. Chen et al. [9] and Yu et al. [10] studied the influence of process parameters on coating cracks, and on this basis, they found the optimal parameter combination without cracks through orthogonal experiments and intelligent algorithms. The research by Chen et al. [11] and Wang et al. [12] shows that the use of rare earth elements and auxiliary fields can refine the microstructure and reduce stress concentration in the coating. Cai et al. [13] and G. Bidiron et al. [14] demonstrated that the use of the transition layer and the preheating of the substrate can effectively reduce the thermal expansion difference in the coating, thereby reducing the generation of thermal stress. With the continuous innovation of technology, the existing equipment can measure the residual stress in coatings, but the measurement process is complex and costly. To enhance the efficiency of crack prevention measures, numerical simulation provides researchers with new methods. Abdelkrim Bouabbou et al. [15] simulated the interaction between powder and laser during the selective laser melting (SLM) process to reveal the influence of multi-physics field coupling on defect evolution. Zhao et al. [16] established a finite element model based on the properties of shaft materials, analyzed the distribution of residual stress in the temperature field and stress field, and simulated the solidification and melting processes of the molten pool in the flow field. In addition, the influence of process parameters on coating defects is also summarized. With the maturity of the integrated technology of process–material–simulation, the above-mentioned defect mitigation strategies have gradually been applied in fields such as equipment maintenance. Olivia Kendall et al. [17] conducted a repair study on high-carbon steel rails through laser cladding, tempering treatment, and grinding techniques. After cladding, after tempering treatment at 540 °C, the tensile stress of the coating was significantly reduced, and the martensite in the heat-affected zone completely disappeared, making the hardness of the substrate and the rail more compatible. This treatment method also maintains the compressive stress on the surface of the cladding layer, which helps improve the wear resistance and rolling contact fatigue life of the rail. Yu et al. [18] conducted a thermist-force coupling simulation using ANSYS 13.0 software, optimized the repair parameters of the planetary gear reducer, and they verified its effectiveness in the experiment, solving the failure problem of gears caused by cracks.

In laser cladding, apart from improving and preventing cracks, enhancing the performance of the coating is also crucial. Therefore, this paper introduces the influence of laser cladding (iron-based, nickel-based, cobalt-based, and ceramic) materials on the coating from Section 2; Section 3 introduces the optimization of laser power, laser spot diameter, powder feeding method, scanning speed, and process parameters; Section 4 introduces the classification of laser cladding cracks, the causes of cracks, and the methods for improving and preventing cracks in coatings. Finally, it looks forward to the future trends of laser cladding.

## 2. Laser Cladding Composite Coating Material System

The selection of cladding material is a critical factor influencing the performance of the coating. When the material is selected, it should exhibit good solid-state fluidity and processing performance to ensure a uniform powder feeding process. At the same time, the cladding material should offer desirable physical properties, such as good wettability, a similar thermal expansion coefficient, and thermal conductivity, to reduce the generation of thermal stress and residual stress. This also helps improve the macrostructure of the cladding coating, making it denser and smoother. Currently, the cladding materials typically consist of metal powders and ceramic powders. In metals, iron-based, cobalt-based, and nickel-based self-fusible alloy powders are commonly used [19]. These powders should contain elements such as boron (B) and silicon (Si), which help in self-deoxidation and slag formation. When the laser beam melts the cladding material, the oxidation reaction is preferred, resulting in the formation of borosilicate on the surface of the melt pool. This process reduces the oxygen content and inclusions in the coating so that the liquid metal in the melt pool and the matrix are better combined.

### 2.1. Iron Base Self-Fusing Alloy Powder

Austenitic stainless steel and high-chromium cast iron are common self-fusing powders for iron-based alloys. These materials are often used for workpieces that are easily deformed and require localized wear resistance, and they offer good adaptability when cast iron and carbon steel are used as the base material. Suitable amounts of B and Si slag-forming elements are added to austenitic stainless steel alloy powder to reduce the crack formation and improve the plastic toughness of the coating. When B and Si are added to the high-chromium cast iron alloy powder, M_7_C_3_, M_3_C_2_ carbides, and M_2_B borides are easily formed due to their high content of C and Cr. These hard carbides enhance the hardness and wear resistance of the alloy coating [20]. Ouyang et al. [21] prepared an iron-based alloy coating with a thickness of approximately 1.2 mm on the surface of 27SiMn steel. Due to the presence of carbides M_7_C_3_, borides M_2_B, and Cr_3_Si in the coating, the cladding layer exhibited superior corrosion resistance compared to the substrate. Hua He et al. [22] performed laser cladding using an Fe-C-Si-B alloy on the surface of U71Mn rail steel to obtain a cladding layer with a typical hypoeutectic structure and in situ carbide particles. Tribological tests reveal that the microhardness of the laser cladding layer reaches HV 750–800, and its wear resistance is improved by a factor of eight.

### 2.2. Nickel-Based Self-Fusing Alloy Powder

Nickel-based alloy powders are commonly used for workpieces with high localized resistance to heat corrosion and wear. Compared with iron-based materials, nickel-based alloys exhibit superior oxidation resistance, impact resistance, wear resistance, corrosion resistance, and high-temperature self-lubricating properties, making them highly effective for the repair and reinforcement of components. Wan et al. [23] prepared 0.7 mm-thick nickel-based and iron-based composite coatings on the surface of 40Cr steel. X-ray diffraction (XRD) analysis revealed that the nickel-based coating is primarily composed of a γ-(Ni, Fe) solid solution, FeNi_3_ and Ni_31_Si_12_ eutectic phases, and hard chromium borides (CrB) and carbides (Cr_7_C_3_). In contrast, the iron-based coating mainly consists of austenite and (Fe, Cr)_7_C_3_ hard particles. The nickel-based coating demonstrates significantly higher hardness (960 HV0.3), along with superior corrosion and wear resistance compared to the iron-based coating (357.6 HV0.3). Ni-based alloy powder contains nickel, carbon, chromium, silicon, boron, and other elements, easy-to-generate carbide, boride, and nitride, and other hard phases in the coating process, thereby significantly enhancing the coating’s hardness and wear resistance. Zhang et al. [24] prepared Ni60-Ti_3_SiC_2_ coatings on the surface of S355 steel, in which the microstructure of Ni60 coatings showed massive and dendritic crystals, and the microstructure of the coatings was transformed into fine-grained crystals when 5% Ti_3_SiC_2_ was added. XRD analysis showed that the coatings generated γ-Ni, Cr_7_C_3_, TiC, Fe_2_C, and Ti_3_SiC_2_ phases. Among them, Cr_7_C_3_, TiC, and Fe_2_C are hard phases whose presence significantly enhances the hardness and wear resistance of the coatings.

### 2.3. Cobalt-Based Self-Fusing Alloy Powders

Cobalt-based coatings are commonly used for workpieces requiring high hardness, wear resistance, and fatigue resistance. Compared with nickel-based powders, the prepared fusion cladding exhibits excellent corrosion resistance, wear resistance, and high-temperature oxidation resistance. The alloy powder contains mainly Co, Cr, Fe, Ni, and C elements. Fang et al. [25] prepared Ni-based and Co-based coatings on the surface of 316L stainless steel using laser cladding technology. The Ni-based coatings were primarily composed of γ-(Fe, Ni), Cr_7_C_3_, and Ni3Si phases, with a microstructure consisting of equiaxed and dendritic crystals, while the Co-based coatings were primarily composed of γ-Co, Cr_7_C_3_, and Co_7_W_6_ phases, exhibiting a microstructure characterized by columnar and dendritic crystals. The presence of equiaxial crystals in Ni-based coatings contributed to a more uniform microstructure distribution and resulted in slightly higher hardness compared to the Co-based coatings. However, the oxide layer in the Co-based coatings contains Fe_2_O_3_, Cr_2_O_3_, NiO, WO_3_, and Co_3_O_4_, which enhances the wear resistance and corrosion resistance of the Co-based coating. Liu et al. [26] prepared ST6-Co alloy coatings on the surface of H13 steel. The results showed that the coating was primarily composed of the γ-Co phase (face-centered cubic) and the M_23_C_6_ eutectic phase, where M represents Cr and Fe. The solid solution strengthening of nickel, chromium, and silicon in the eutectic and the diffuse strengthening of carbides increased the hardness of the coating by 3.55 times.

### 2.4. Ceramic Powder

#### 2.4.1. Types and Advantages of Ceramic Powders

Ceramic materials offer several advantages, including high strength, stable chemical properties, and resistance to heat and vibration. They are commonly used to enhance the surface hardness, wear resistance, and high-temperature oxidation resistance of components. At present, the commonly used ceramic powders are silicide ceramic powder (MoSi_2_) [27], oxide ceramic powders (Al_2_O_3_, ZrO_2_, TiO_2_) [28,29,30], and carbide ceramic powders (WC, Cr_3_C_2_, B4C) [31,32,33]. In laser cladding, the formation of ceramic phases plays a crucial role in enhancing the hardness and wear resistance of the coating.

When the ceramic powder in the cladding material is insufficient, the improvement of the coating performance is not obvious. When the ceramic powder is excessive, the molten pool exhibits poor wettability and low fluidity, and the distribution of the ceramic phase is uneven, causing segregation and stress concentration. These factors contribute to increased coating brittleness, making it more susceptible to cracking. Therefore, incorporating an appropriate number of ceramic particles is essential for optimizing the coating’s overall performance. Liu [34] et al. investigated the effect of WC particle content on the coating properties of Ni50. The results showed that, with increasing WC content, the coating structure becomes finer and denser. The formation of hard phases such as WC, W_2_C, M_23_C_6_, and M_7_C_3_ significantly enhanced the hardness and wear resistance of the coating. When the WC content reached 20 wt%, the coating hardness increased to 817.9 HV.

#### 2.4.2. The Introduction Method for the Ceramic Phase

When preparing metal composite coatings with ceramic phases, two methods are usually employed: the direct addition method and the in situ synthesis method [35]. The direct addition method requires low energy, but ceramic particles are large and susceptible to defects such as cracking, which limits their effectiveness in enhancing coating performance. In contrast, the in situ synthesis method is achieved by chemically reacting the ceramic composite powder under laser irradiation, which is precipitated during a rapid solidification process and diffusely distributed in the coating. The ceramic phases produced via this method are strongly bonded to the metal, featuring finer and more uniform particle distribution. Wang et al. [36] generated a composite coating on the surface of Inconel 718 alloy using a mixture of Ni60A and WC powders. The results show that, under the irradiation of the laser, the C element decomposes from the WC particles and reacts with the Ni, Cr, Fe, and Nb elements in Ni60A to form the NbC, (Ni, Cr, Fe) 23C6 enhanced phase. These uniformly distributed reinforcing phases avoid the problem of stress concentration caused by excessive concentration of local reinforcing phases, thus enhancing the overall coating performance. Li et al. [37] prepared Ti(C, N) ceramic composite coatings on TC4 titanium alloy substrates using TiN, pure Ti powder, graphite, and In625 powder. The results show that TiN and graphite undergo a displacement reaction when the temperature is higher than 1890 K, generating the Ti(C, N) phase. The in situ generated TiC, Ti(C, N), and Ti2Ni play a structural role in the coating as a “skeleton”, which hinders the movement of the incision tear point and improves the wear resistance of the coating.

#### 2.4.3. The Optimization Method of the Ceramic Phase

The differences in physical properties, such as the coefficient of thermal expansion, between ceramic powder and the base material can lead to poor interfacial bonding during cladding, making the resulting coating prone to cracking. To mitigate residual stresses and prevent cracks, several methods are commonly employed, including preheating and post-heating, nano-ceramic particles, the optimization of process parameters, rare earth elements, and gradient coatings.

**Figure 1:**
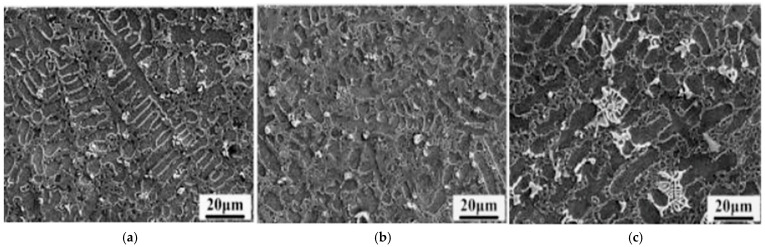
Effect of different nano-WC incorporation on the organization of the fused cladding layer. (**a**) 0.0 wt.%, (**b**) 3.0 wt.%, and (**c**) 6.0 wt.% [39].

When nano-ceramic particles are introduced into laser cladding, the ceramic particles act as heterogeneous nucleation sites in the molten pool, promoting grain nucleation in the cladding layer and inhibiting grain growth, thereby resulting in grain refinement [38]. Moreover, the nano-ceramic particles can optimize molten pool dynamics, enabling uniform distribution of the particles in the cladding layer, thus reducing the stress concentration and thereby reducing the tendency of cracking and improving the comprehensive properties of the coating. Li et al. [39] prepared Ni45/WC coatings on the surface of 42CrMoA alloy steel using a 5 kW CO_2_ laser. As shown in Figure 1, with the increasing addition of nano-WC, the microstructure was first refined and then coarsened, and the coating organization was the finest when 3.0 wt.% was used. This effectively relieves stress concentration and reduces cracks. Both hardness and wear resistance were improved compared to the unmodified nano-WC coating.

**Figure 2:**
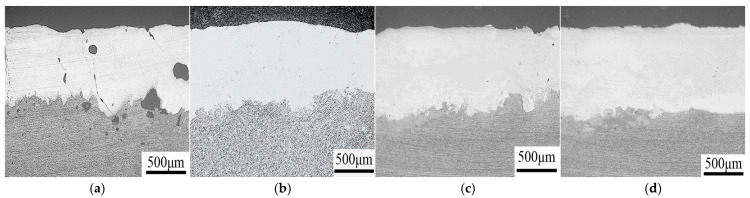
Surface morphology of coating: (**a**) Ni60, (**b**) La2O3Ni60, (**c**) Y2O3Ni60, and (**d**) CeO2Ni60 [42].

The incorporation of rare earth elements in laser cladding improves the mobility of the molten pool, reduces the surface tension of the molten metal during the coating formation process, improves the wettability at the solid–liquid interface, and thereby promotes the uniform formation of the cladding layer. Rare earth elements exert a “pinning effect” within the grain boundaries, restricting the growth of grains and blocking crack propagation by multiple grain boundaries, which contributes to improved fracture toughness. Furthermore, rare earth elements readily combine with oxygen and impurity elements to generate high-melting-point compounds, avoiding the formation of pores and inclusions [40]. Zhang et al. [41] TiC reinforced Ti matrix composite coating prepared on the surface of Ti6Al4V alloy. The results indicated that the addition of Y_2_O_3_ reduces the cooling rate of molten pool and increases the heat input, which makes TiC dendrites more developed and causes a large number of tertiary dendrites to appear. The addition of Y_2_O_3_ also caused Ti_2_Ni to disappear and Cr1.93Ti1.07 form, significantly improving the formation quality of laser cladding coating by eliminating cracks and greatly reducing porosity. Wang et al. [42] prepared rare earth oxide cladding layers on the surface of 6063 aluminum alloy. As shown in Figure 2, the addition of rare earth oxides makes the coating surface smoother, and no porosity cracks occur. The cladding layer exhibited dense dendrites, the grains are significantly refined, and the hardness is increased from 200 HV to 350 HV. Notably, the hardness of the cladding layer did not decrease with increasing depth.

**Figure 3:**
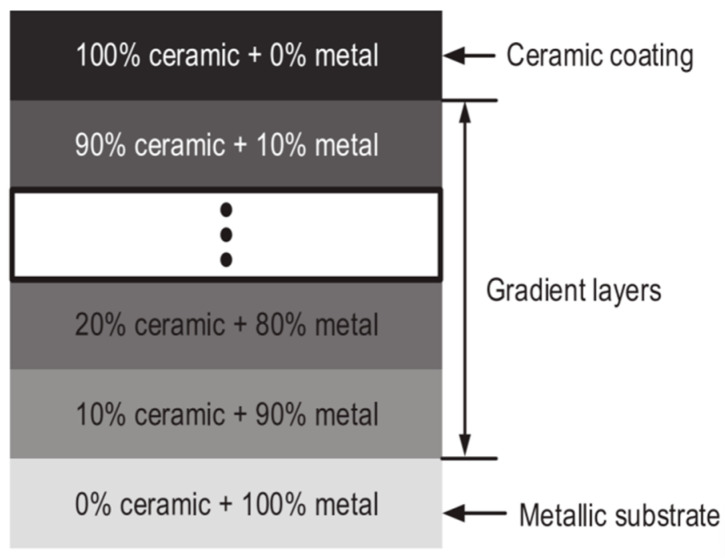
Ladder diagram structure diagram [45].

When high concentrations of hard particles are introduced into the coating, the improvement effect of using rare earth elements and nano-ceramic particles on coating defects is limited. After conducting a large number of experiments, researchers designed coatings with gradient changes in performance by regulating the composition of the cladding material, the number of coating layers, and the thickness. The structure of this layer is shown in Figure 3. An increased ceramic content in the coating mitigates the performance discontinuity at the cladding substrate interface, thereby enhancing the matching between adjacent layers. This trapezoidal coating reduces the thermophysical difference between the substrate and the material, and it also decreases the thermal stress and residual stress of the coating. Wang et al. [43] prepared WC gradient composite coatings from low concentration to high concentration, effectively solving the problems of mismatch between the substrate and the coating and high crack sensitivity in high-concentration hard particles. Lin et al. [44] fabricated gradient TiB_2_/TiB coatings on titanium alloy substrates. The results show that the use of gradient coatings reduces stress concentration in the coating and improves the fracture toughness in the middle and bottom of the coating. This improvement in performance is mainly attributed to the debonding of TiB_2_ particles and the excellent crack-arresting ability of TiB short fibers. These characteristics contribute to crack deflection, thereby enhancing the overall mechanical properties of the coating.

In conclusion, when selecting materials, a comprehensive consideration should be directed to the physical and chemical properties of the materials, their compatibility with the substrate, and their economy. This is to enhance the bonding strength between the coating and the substrate while reducing defects in the coating. In terms of improving the performance of the coating, methods such as introducing nano-ceramic particles, rare earth elements, and trapezoidal composite coatings can refine the coating structure, reduce stress concentration, and thereby effectively suppress the generation of cracks.

## 3. Influence of Process Parameters on the Cladding Layer

Laser cladding is a process of dynamic melting and rapid solidification, in which chemical reactions between various elements and physical phenomena such as heat conduction and heat convection between materials frequently occur. In addition to the performance differences between materials that affect the performance of the coating, the manipulation of process parameters can effectively regulate the temperature field, thus affecting the microstructure structure and forming quality [46]. At present, the main process parameters are laser power, the spot diameter, the scanning speed, powder feeding mode, and protective gas, among others.

### 3.1. Laser Power

Laser power exerts an important influence on the bonding between coating and substrate, the microstructure, and the physical properties of the cladding layer. Excessive heat input leads to the sintering of cladding powder and exacerbates the cracking in the cladding layer. Conversely, when the heat input is insufficient, the powder fails to melt completely, resulting in problems such as the spheroidization of the coating surface and gaps at the joints. Therefore, selecting appropriate laser parameters and changing the laser density can effectively suppress defect generation. Wang et al. [47] laser-coated Stellite12 coating on the surface of H13 steel. It was found that there were unmelted powder and obvious cracks on the surface of the coating at lower power. At higher laser power, the heat-affected zone (HAZ) on the surface of the substrate becomes larger, and cracks exist inside. The increase in laser power also caused the Fe element to diffuse into the coating, and the dilution rate gradually increased. When the power was 2200 W, the surface of the coating was well formed, with no obvious internal defect, and the mechanical properties of the coating were optimized. Cai et al. [48] prepared Ni_3_Al-based alloy cladding layers with varying laser powers. The findings showed that, as the laser power increased, the dilution rate of the coating increased from 4.12% to 8.87%, the carbide particles in the coating grew continuously, and the average size increased from 1.51 µm to 2.23 µm. The wear resistance of the coating initially increased and then decreased, and the coating was best when the laser power was 2 KW.

### 3.2. Laser Spot Diameter

The spot diameter is a critical process parameter, which has a direct impact on the molten pool geometry, temperature distribution, and coating quality. The spot diameter plays a crucial role in determining the aspect ratio of the coating. The increase in the spot diameter raises the total heat input, but due to the dispersion of energy density, the temperature gradient inside the molten pool is lowered, which helps lower the thermal stress within the coating. However, the dispersion of energy density can also make the depth of the melt pool shallow, leading to a non-uniform internal structure in the coating and ultimately impacting the performance of the coating. When the spot diameter is reduced, a finer grain structure can be obtained. However, smaller spot diameter energy concentration may lead to higher thermal stress and a higher risk of cracking. Therefore, selecting a suitable spot diameter can improve the performance of the coating. Chen et al. [49] analyzed the performance test of Ti6Al4V ceramic coatings with different process parameters. The results show that spot diameter is one of the important factors affecting coating quality. The spot diameter not only can control the coating width, thickness, and height difference, thereby reducing the difficulty of subsequent processing, but also can be combined with other processes to improve the hardness and overall performance of the coating. Moreover, some studies have indicated that using large spot sizes and non-circular spot shapes (rectangle, oval) can also improve the coating quality [50].

### 3.3. Powder Delivery Method

**Figure 4:**
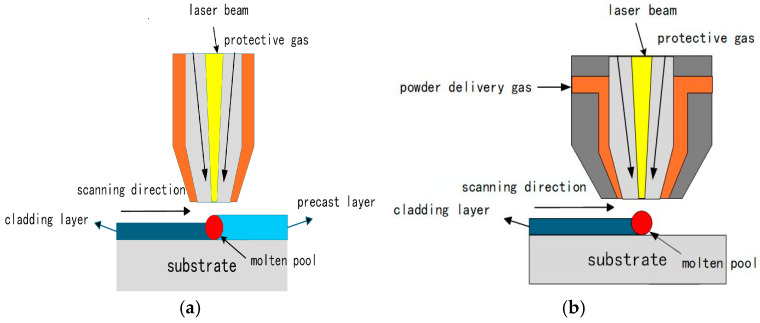
(**a**) Pre-placed powder method; (**b**) coaxial powder feeding method.

In laser cladding, according to the different powder processing methods, the prefabricated powder method and the coaxial powder feeding method are mainly used, as shown in Figure 4. The prefabricated powder method involves directly laying the powder on the surface of the matrix, and the prefabricated layer can be scanned by the laser beam, allowing the powder and the surface of the substrate to melt at the same time and quickly solidify to form a cladding layer. This process is both simple and efficient. The coaxial powder feeding method delivers powder from the feeder into the workpiece surface’s irradiation area under the protection of the inert gas to facilitate material melting and subsequent deposition. The coating produced through this method exhibits high quality and is easier to control automatically. In the coaxial powder feeding method, optimizing the nozzle design ensures stable and uniform powder delivery, improving powder utilization and significantly enhancing coating quality. Su et al. [51] used a CCD experimental design and a BP neural network optimized through a genetic algorithm to optimize the internal structural parameters of the nozzle, while the external protective gas parameters were optimized using an orthogonal experimental design. The data reveal that, after optimization, with powder aggregation, the diameter of the nozzle was reduced to approximately 2.71 mm, and the powder aggregation focus distance from the nozzle outlet was about 10 mm, increasing the powder utilization rate to about 57%. Furthermore, studies on the powder delivery rate have shown that a well-controlled powder feeding rate reduces the risks of particle buildup, thermal non-uniformity, and over-sintering. Through experiments, Zhang et al. [52] concluded that an appropriate powder feeding rate can control the coating aspect ratio and dilution rate, while the rapid cooling of the molten pool restricts the time available for grain coarsening, promotes grain refinement, and ultimately enhances the coating’s hardness.

### 3.4. Scan Speed

Scanning speed has a similar impact on coating effects as laser power. A low scanning speed increases the time spent in the melt pool, providing ample time for the cladding microstructure to grow, which ultimately reduces the coating’s properties. Conversely, a high scanning speed results in the powder absorbing too little energy and not melting completely, which results in inadequate bonding between the cladding and the substrate. Therefore, selecting an optimal scanning speed based on the material properties is crucial for improving coating performance. Li et al. [53] studied the effect of scanning speed (3 mm/s–9 mm/s) on the dilution rate, wear resistance, and hardness of Ti/TiBCN fused cladding layer on Ti6Al4V substrate. The experiment demonstrated that the hardness of the coating with scanning speed, increasing initially and then decreasing, and the wear decreases and then increases. At a scanning speed of 7 mm/s, the coating had the finest microstructure and the fewest defects. Jiao et al. [54] studied the cladding of T15 M high-speed steel powder on Q235 substrates at different scanning speeds (100, 200, and 300 mm/min). It was concluded that, as the scanning speed increases, the carbides increase, the grain size becomes larger, and the coating is prone to producing cracks and pores. A low scanning speeds lead to excessive segregation. At a scanning speed of 200 mm/min, the coating revealed the most significant fine-grain strengthening and optimal performance.

### 3.5. Optimization of Process Parameters

The optimization of process parameters is multifaceted. Currently, common methods include the single-factor experiment method, orthogonal experimental design, response surface methodology, and intelligent optimization algorithms. The single-factor experiment method involves altering a single process parameter and observing its impact on coating performance, the orthogonal experimental design systematically examines the influence of multiple factors on coating performance, the response surface methodology constructs mathematical models to analyze the relationships between process parameters and coating performance, intelligent optimization algorithms utilize particle swarm optimization (PSO), the Non-Dominated Sorting Genetic Algorithm II (NSGA-II), and other techniques to simultaneously optimize multiple objective functions, identifying the optimal solution set. Because single-factor optimization has limited effectiveness in improving coating quality and reducing defects, multiple process parameters are often optimized in practical experiments. Table 1 summarizes the optimization of process parameters, where laser power is denoted as P, scanning speed as V, the powder feed rate as Vf, the dilution rate as η, the aspect ratio as W/H, the contact angle as θ, and the overlap rate as Φ.

When several optimized process parameters are limited and their relationship with coating performance is approximately linear, the orthogonal experimental design method can be employed to identify optimal parameters while minimizing the number of experiments. Yang et al. [62] optimized the laser cladding process parameters (laser power, scanning speed, and powder feeding rate) by using the orthogonal test method, reducing the segregation phenomenon of carbon element in the coating, thereby decreasing the number of cracks. On this basis, Dong et al. [63] combined the orthogonal experimental design with variance analysis to demonstrate that optimizing process parameters can significantly enhance coating performance. When interacting and optimizing with multiple factors and having a nonlinear relationship with the performance of the coating, the response surface design method optimizes parameters through experimental design and model fitting, and the intelligent optimization algorithm iteratively searches for the optimal solution, thereby improving data accuracy. Li et al. [56] optimized the parameters for Ni60PTA alloy powder on 45 steel using the response surface methodology (RSM) and identified the optimal combination (laser power of 1477 W, a scanning speed of 5 mm/s, and a powder feeding rate of 17.5 mg/s). Under these conditions, the coating exhibited high quality, and it was free from defects such as cracks, deformation, and porosity. The prediction errors between the model and the experimental results were all below 10%, indicating that the developed model possessed relatively high predictive accuracy. Similarly, Liang et al. [59] employed a random forest regression (RFR) model optimized via the Osprey Optimization Algorithm (OOA), in combination with the Unified Non-Dominated Sorting Genetic Algorithm III (U-NSGA-III), to optimize the process parameters for the in situ synthesis of TiB_2_/TiC particle-reinforced composite coatings via laser cladding. The optimal process parameters were identified as follows: laser power of 1587 W, a scanning speed of 11 mm/s, a powder feeding rate of 1.3 r/min, an overlap rate of 50%, and the content of the Ti and B4C mixed powder of 11%. Under these optimized conditions, the in situ generated TiB_2_ and TiC particles acted as second-phase strengthening agents, significantly enhancing the hardness and wear resistance of the coating. Moreover, the prediction error was less than 3%, further validating the accuracy and effectiveness of the intelligent optimization algorithm.

In addition to the optimization and prediction of process parameters, environmental factors during actual manufacturing (such as powder moisture content, workshop temperature, and ambient airflow) are often unavoidable and may compromise the expected coating performance. Previous studies have shown that excessive moisture in the powder can lead to defects such as agglomeration, incomplete melting, and increased porosity [64]. Therefore, it is critical to thoroughly dry and properly store the powder prior to experimentation. To mitigate the negative influence of external variables on process consistency, researchers have implemented real-time monitoring strategies. Commonly employed techniques include infrared thermography coupled with image analysis software, temperature sensors, and plasma spectroscopy [65]. Infrared cameras and image analysis allow for the evaluation of cladding quality by tracking parameters such as the molten pool temperature, geometry, and cooling rate. Temperature sensors provide real-time data on the thermal distribution within the processing zone, offering direct insights into the powder substrate interaction. Plasma spectroscopy, on the other hand, captures atomic or ionic emissions generated during laser ablation, enabling the analysis of material behavior in the frequency domain. The integration of these monitoring tools, alongside optimized process parameters, facilitates the production of high-quality coatings.

In summary, the application of laser cladding process parameters significantly influences energy density, the melt pool duration, the coating dilution rate, the coating aspect ratio, and the powder utilization efficiency. These factors collectively influence the temperature field distribution, ultimately affecting the quality of the coating. Furthermore, the use of single-factor experiments often fails to significantly improve cladding quality. Consequently, contemporary research and applications frequently employ an orthogonal experimental design or advanced optimization algorithms, such as response surface methodology and genetic algorithms, for multi-parameter optimization. This approach facilitates the identification of optimal parameter sets, thereby minimizing defect formation and enhancing coating quality. Therefore, the optimization of process parameters is essential to achieving high-quality, high-efficiency, and cost-effective laser cladding technology.

## 4. Problems with Laser Cladding and Improvement Measures

### 4.1. Classification of Cracks

**Figure 5:**
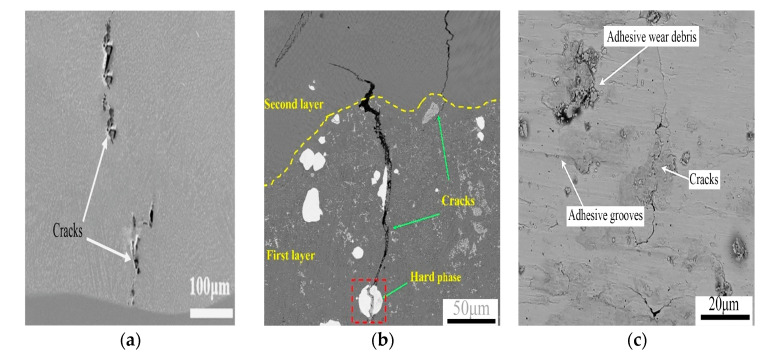

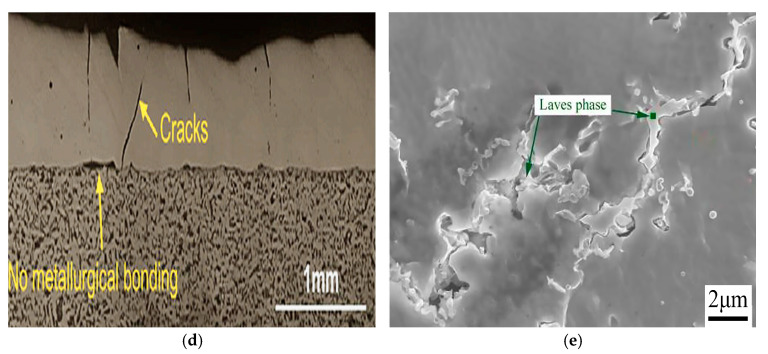
Different crack forms: (**a**) hot crack [66], (**b**) cold crack [67], (**c**) cladding crack [68], (**d**) interfacial matrix crack [69], and (**e**) Lap crack [70].

During the laser cladding process, cracks occur when the cladding layer is affected by stress and its own brittleness, resulting in the destruction of the bonds between atoms within the cladding layer and thus forming gaps. Cracks can be classified into hot cracks and cold cracks according to the temperature, morphology, and location at which they occur [8]. Hot cracks (Figure 5a) mainly occur above the solidification temperature line, distributed at the center of the molten pool, the arc closure of the weld seam, or between dendrites. These cracks typically exhibit high-temperature intergranular fracture characteristics, and the fracture surface has an oxidized color and no metallic luster. Cold cracks (Figure 5b) primarily form below the solidification temperature line, with typical distribution near the fusion line, within the heat-affected zone, or extending into the substrate. The fracture surface is smooth and has a metallic luster. According to the location where the cracks occur, they are classified into cladding layer cracks, interface matrix cracks, and lap cracks. The cracks in the cladding layer (Figure 5c) are caused by factors such as the thermal stress resulting from the solidification and rapid cooling of the liquid metal, the microstructure stress caused by phase transformation, uneven microstructure distribution, and porosity. When the crack is inside, it manifests as an obvious, continuous crack phenomenon along the depth direction. Interface matrix cracks (Figure 5d) are caused by an excessive difference in thermal expansion coefficients between the cladding material and the matrix, as well as uneven temperature and stress distribution in the molten pool, resulting in incomplete bonding between the matrix and the cladding. Lap cracks (Figure 5e) occur at the junction of the lap joint and the base material. Due to the unreasonable selection of the lap ratio, heat accumulation and corresponding variation superposition occur at the lap joint, generating the laves brittle phase. Its formation reduces the toughness of the material, thereby leading to the formation of cracks.

### 4.2. Causes of Cracks in Laser Cladding

Laser cladding is a critical technique for repairing and enhancing components; however, crack formation remains a significant challenge. During the cladding process, the laser energy density is concentrated, creating an inhomogeneous temperature field within the cladding layer. This leads to the development of internal stresses, which, if exceeding the plastic strain limit of the cladding layer, can result in cracking. Such defects significantly degrade coating performance and may even render the component unusable [8]. During the laser cladding process, the internal stresses can be categorized into three types according to on their origin: (1) thermal stress—due to the different coefficients of thermal expansion of different materials, the contraction rate of the cladding layer is also different when it expands thermally and cools rapidly, as is the extrusion stress generated via the difference in the shrinkage rate; (2) microstructural stress induced via phase transformations that occur as the molten pool solidifies and cools; and (3) constrained stress—during cooling, due to the differences in solidification time and volume shrinkage between the liquid and solid phases, the coating is constrained by the surrounding substrate, resulting in constrained stress. Among them, thermal stress is the main factor causing cracks. In addition, for materials undergoing isotropic transformation, microstructural stresses play a crucial role in the overall internal stress state.

#### 4.2.1. Influence of Internal Stress on Coating

Due to the different thermal expansion coefficients of the cladding material and the base material, the material will undergo thermal contraction in opposite directions during the heating and cooling stages. The interface is forced to “stretch” and “compress” simultaneously, resulting in a temperature gradient during the cladding process and thus generating thermal stress. When the thermal stress exceeds the yield limit of the material, cracks will appear in the coating [71]. The formula for thermal stress is as follows.

(1) $$\sigma_{T}=\frac{ET_{1}(\alpha_{1}-\alpha_{2})}{1-V_{1}}\geq \sigma_{\max}$$

where σ_T_ is the thermal stress, E is the modulus of elasticity of the cladding material, α_1_ is the thermal expansion coefficient of the cladding material, α_2_ is the thermal expansion coefficient of the base material, T_1_ is the temperature difference between the cladding layer and the outside world, and v_1_ is the Poisson’s ratio of the cladding layer. The equation reveals that the thermal stress magnitude is determined by both the thermal expansion coefficient differential between cladding and base materials, and the outside world affectsf the magnitude of the thermal stress. When α_1_ < α_2_, it shows tensile stress in the cooling stage and compressive stress in the heating stage. When α_1_ > α_2_, it shows compressive stress in the cooling stage and tensile stress in the heating stage. Among them, tensile stress enhances crack sensitivity, and compressive stress can effectively alleviate crack propagation [72]. Additionally, researchers categorize these systems into metal–metal and ceramic–metal types based on cladding and base material combinations. Cracks can be significantly mitigated when the thermal expansion coefficient is ≤5 × 10^−6^ K^−1^ for metal-metal systems, or ≤8 × 10^−6^ K^−1^ for ceramic–metal systems [73,74,75]. Therefore, the selection of materials will directly affect the performance of the coating.

#### 4.2.2. The Influence of Residual Stress on Coatings

**Figure 6:**
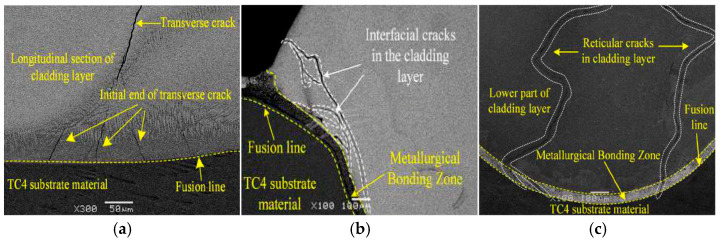
(**a**) Transverse cracks, (**b**) longitudinal cracks, and (**c**) mesh cracks [76].

When the external load or temperature field is non-uniform, resulting in plastic deformation of the fusion cladding layer, after the external load is removed or the temperature field is normalized, residual stresses will persist internally. We define these as residual stresses. Residual stress is the combined result of thermal stress, phase transformation stress, constraint stress, etc. When the residual stress exceeds the fracture limit of the material, cracks will occur in the coating. Gao et al. [76] prepared FeCoCrNi fusion-coated coatings on the surface of TC4 titanium alloy and observed that the formation of three types of cracks were induced via stress in the coating: transverse cracks normal to the scanning direction, longitudinal cracks parallel to the scanning direction, and networked cracks forming grid-like patterns. As shown in Figure 6a for transverse cracks, the cracks were generated via the confining stress in the scanning direction. They initiated at the bond between the coating and the substrate, and they extended perpendicularly into the coating, with the maximum value of stress near the fusion line. Longitudinal cracks are shown in Figure 6b. The cracks resulted from tensile stresses in the cross-section of the coating. They originated at the surface of the coating, and they extend from the surface of the coating along the bonding zone to the bottom, exhibiting characteristics of through-crystal fracture. When the cracks in the coating develop in multiple directions, as shown in Figure 6c, they are reticular cracks. The cracks are driven by thermal stresses, occur within the coating, and are characterized by both along-crystal and through-crystal extension. Shi B et al. [77] investigated the effect of process parameters on cracking. The results revealed that the powder feeding speed and power also had a great influence on the crack generation. When the powder feeding speed is too large or the laser power is small, the energy delivered per unit mass of powder is small, and this energy deficiency results in the formation of severely inhomogeneous CrB, Cr_7_C_3_, and Cr_23_C_6_ hard phases in the coatings, which increases the residual tensile stresses and effects heightens the cracking susceptibility of the Ni60A coating.

#### 4.2.3. Effect of Other Factors on Coatings

During fusion cladding, disparities in physical properties and chemical incompatibility between materials result in excessive internal stresses, leading to a high sensitivity to cracking for the coating. Impurities and pores in the fusion cladding are also causes of cracking. Laser cladding is characterized by rapid solidification in the self-fusing alloy powder, etc. Due to the deoxidation and slagging elements after the reaction has failed to reach the surface of the cladding layer in its entirety, part of the cladding layer in the formation of entrapment slag results in stress concentration, thus generating cracks. Li et al. [78] found that, in the preparation of Ni50A-WC coating, CO and CO_2_ in the coating failed to escape in time due to too-fast curing. Porosity was generated in the coating. Meanwhile, the microstructure of the coating primarily consists of a matrix phase and a significant amount of second phases. These second phases can be categorized as either granular or massive. The granular second phases are predominantly composed of carbides, while the massive phases contain both borides and carbides. Although the formation of second phases contributes to the strengthening of the coating, an excessive amount of these phases can induce high internal stresses at the interface between the matrix and the precipitated phases, which ultimately lead to cracking. Yu et al. [73] significantly mitigated transverse and longitudinal cracking in nickel-based coatings by fabricating an austenitic stainless steel mesh on the substrate surface, thereby reducing thermal expansion coefficient disparities. However, there is a gap between the austenitic stainless steel mesh and the substrate, and during rapid solidification, there is still a residue of gas, which forms pores and improves the crack sensitivity.

### 4.3. Crack Prevention and Control Measures for Laser Cladding

In the laser cladding process, residual stresses accumulate over time and ultimately lead to crack formation. At present, the factors influencing the residual stress mainly include material physical properties, the uniformity of the temperature field, phase-change stress, and pore entrapment. To prevent crack formation, five measures can be taken: The first one is the selection of processes in the preparation stage, which reduces the physical differences between the cladding material and the substrate, improves the energy distribution in the molten pool, and ultimately achieves the regulation of the residual stress level and distribution. The second type is the application of auxiliary scenarios during the cladding process to improve the temperature distribution and microstructure distribution of the molten pool and reduce defects in the coating. The third method uses heat treatment technology to release residual stress in the coating. The fourth method is to use auxiliary software to predict residual stress and reduce the generation of residual stress through optimization. The fifth approach is to seek out new processes and structural materials to enhance the performance of the coating.

#### 4.3.1. The Selection of Processes in the Preparation Stage

During the preparation stage, the selection of materials, the setting of reasonable process parameters, and the application of gradient coatings can reduce the defects of the cladding layer after molding. In material selection, materials with good plasticity and similar physical properties to the base material should be prioritized. In addition, the research found that the appropriate addition of other materials can also improve the coating properties while reducing crack sensitivity, for example, materials with negative thermal expansion coefficients (ZrW_2_O_8_, HfW_2_O_8_), rare earth elements, nanoparticles (Al_2_O_3_, WC, TiO_2_), alloying elements, etc. Among them, rare earth elements and nanoparticles reduce stress concentration in the coating by refining the structure. Negative expansion coefficient materials reduce crack sensitivity by lowering the thermal expansion coefficient and elastic modulus of the material [79]. Through research, it was also found that the addition of some alloying elements refines the coating structure, enhances the toughness of the coating, and thereby reduces defects. Table 2 is a summary of the improvement in the performance of alloy coatings.

When preparing a cladding layer containing ceramic particles, the cooling rate of the molten pool is too fast, or the decarburization of ceramic particles during the reaction with oxygen leads to the formation of CO and CO_2_, which are not properly expelled. Extending the cooling time or the addition of an element having a strong affinity for oxygen can reduce the pores in the coating, thereby improving the quality of the coating. Among these, the addition of Ti the element can generate TiC, which generates significant heat and delays the cooling process, thus allowing sufficient time for the gases in the coating to be removed. Additionally, the inclusion of Ti helps disperse local stresses in the coating, enhancing its overall performance [78]. Yamaguchi et al. [85] added an Al element to WC-Co alloy powder, which prevents the formation of CO gas and reduces porosity due to the strong affinity of Al for oxygen and also prevents the decarburization of WC, which produces brittle phases such as W_2_C or M_6_C, thus reducing cracking.

In terms of the use and optimization of process parameters, it can be concluded according to Section 3 that the optimal parameter group can be found through orthogonal experimental design or advanced optimization algorithms (response surface method, genetic algorithm, etc.), thereby reducing the number of experiments and the occurrence of defects in the coating.

In terms of the application of gradient coatings, apart from the improvement of coating defects of high-concentration ceramic particles, as described in Section 2.4.2, they are also widely used in cemented carbides and high-entropy alloys. However, the preparation of trapezoidal coatings also presents the following difficulties: (1) The elemental ratio design of the gradient transition layer needs to take into account both functionality and metallurgical compatibility; otherwise, it is prone to cause defects such as uneven structure and cracks. (2) The laser beam inevitably remelts the previous layer, causing the dilution of elements between layers and thus making it difficult to achieve the gradient functional layer. (3) The expansion coefficients between layers with different gradients vary greatly, which tends to cause hot cracks or delamination cracks. (4) High hard particle content is prone to precipitating brittle compounds at the interface, reducing toughness and bonding strength.

To address the aforementioned problems, researchers have adopted multiple strategies for improving the preparation of gradient coatings. When Zhang et al. [86] prepared the trapezoidal coating, they designed an increase in the powder thickness of each layer by 0.5 mm so that the chemical composition of the coating presented a uniform stepped change and effectively avoided the dilution of elements between layers. Lu Ping et al. [87] studied the angle of the laser melting direction and concluded that, when the interlayer texture angle was 90°, an “L”-shaped structure with a refined microstructure and a uniform distribution was formed. On this basis, Fe-Cr-Ni helical gradient multilayer coatings were prepared to keep the interior of the coating in a state of compressive stress. Sun et al. [88] introduced an intermediate transition layer with 100% Ni content to reduce the difference in the coefficient of thermal expansion between the cladding material (Ni60B powder) and the substrate material (38CrMoAl steel), thereby reducing the crack sensitivity of the coating. Cui et al. [89] prepared FeCoCrNiMnAl0.5 coatings with a lower stacking layer misenergy face-centered cubic (FCC) structure in the lower part and FeCoCrNiMnAl gradient high-entropy alloy (HEA) coatings with a higher stacking layer misenergy body-centered cubic (BCC) structure in the upper part by changing the content of Al. Under high-temperature compression, the lower coating can absorb dislocations, enhancing its resistance to plastic deformation and reducing the tendency of cracks on the coating surface, while the upper coating maintains high strength and performance, ensuring the overall performance of the coating.

#### 4.3.2. Application of Auxiliary Fields in the Cladding Process

In addition to the processes in the preparation stage, the application of auxiliary fields during the cladding process can also enhance the performance of the coating. Auxiliary fields help homogenize the composition of the molten pool and reduce elemental segregation. They also promote an increased nucleation rate, refine the microstructure, and alleviate local stress concentrations, thereby mitigating or even eliminating defects such as cracks and pores in the coating. Commonly applied auxiliary fields include ultrasonic vibration, friction stirring, and electromagnetic fields.

**Figure 7:**
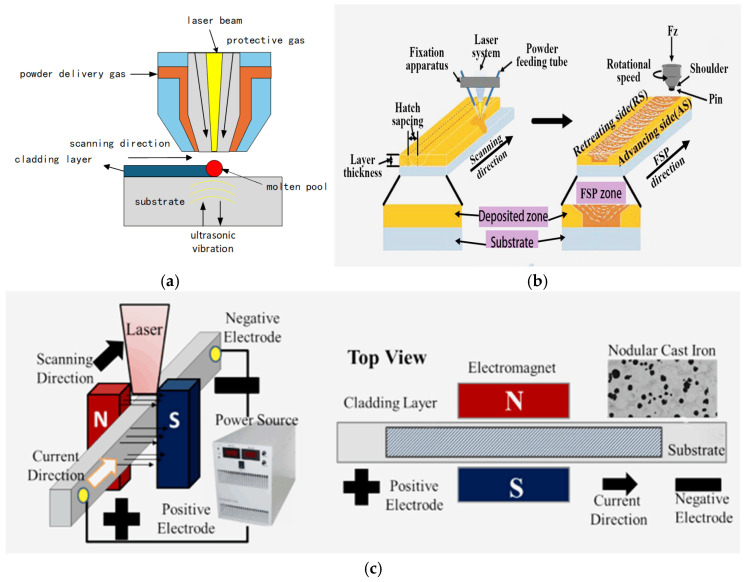
Composite diagram of the auxiliary field: (**a**) ultrasonic vibration composite diagram; (**b**) friction stir laser cladding composite diagram [92]; (**c**) electromagnetic-assisted laser cladding composite diagram [93].

Ultrasonic vibration (Figure 7a) directly acts as the oscillator into the molten pool, using various effect mechanisms, such as cavitation and sound flow, to break down the growing dendrite structure. Meanwhile, the vibration promotes the flow of the melt in the molten pool, causing the broken grains to redistribute and form non-uniform nucleation and thereby significantly increasing the number of nucleation and achieving grain refinement. Wang et al. [90] fabricated an Fe-Ni-Ti composite coating on 316L steel substrate via laser cladding. The research finds that ultrasonic vibration makes the temperature and refined microstructure distribution in the molten pool uniform, reduces the temperature gradient, and decreases the residual stress of single and multiple coatings. In addition, some scholars have studied ultrasonic irradiation technology on the basis of ultrasonic vibration technology. Zhao et al. [91] fused Ni-based WC coatings on Cr12MoV die steel and found that ultrasonic irradiation reduced the surface roughness of the coating. Through cavitation, acoustic flow, and thermal effects, it improved the flow of the molten pool, transformed the coarse columnar crystals in the coating into equiaxial crystals, and converted part of the residual tensile stress in the coating into residual compressive stress, thereby reducing the occurrence of cracks.

**Figure 8:**
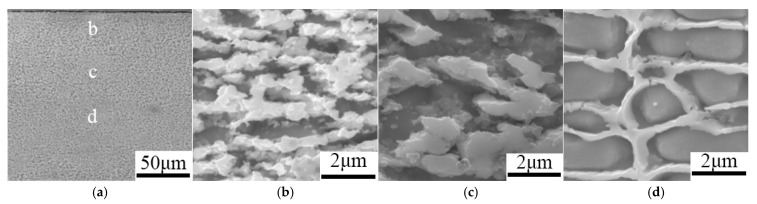
Different depths from the top surface: (**a**) low magnification image, (**b**) enlarged image of the plastic zone, (**c**) zone of thermo-mechanical effect, (**d**) zone of laser fusion coating [94].

Friction stir processing (FSP) (Figure 7b) induces a coupled effect of frictional heat and plastic deformation on the melt pool, refining the microstructure and enhancing the coating’s toughness and thereby mitigating defects such as porosity, cracking, and elemental segregation within the coating. Xie et al. [94] used the stirred friction technique to act with a Ni-Cr-Fe coating and divided the stirred coating, from top to bottom, into three parts, a plastic zone, a thermo-mechanical effect zone, and a laser fusion coating zone. The cracks in the coating were closed in the plastic region. The analysis, as shown in Figure 8, shows that FSP not only refines the particles but also destroys the original reticulation structure and transforms the reticulated carbides into nanoparticles in austenite.

The electromagnetic field (Figure 7c) applies a stable current to the substrate, generating a uniform magnetic flux density distribution between two magnetic poles. This induces accelerated molten pool flow, which promotes grain refinement in the coating. Furthermore, the electromagnetic field reduces the coating’s elastic modulus, enhances its hardness and wear resistance, and simultaneously decreases porosity while suppressing crack formation. Zhai et al. [95] investigated the effects of an electromagnetic field and found that electromagnetic stirring does not change the composition of the phases but affects the distribution and morphology of the phases. It converts γ (Fe, Ni) dendrites into isometric crystals and improves the plasticity of the coating. It disrupts the chain-like structure of the hard phase Cr_7_C_3_, resulting in the formation of separate rod-like structures, increasing the continuity of the matrix and reducing the occurrence of cracks. Crack occurrence decreased by approximately 43% (from 7 to 4 per specimen), while the average coating microhardness increased to 923.7 HV 0.2. Furthermore, when there is a serious mismatch between the substrate and the cladding material, the improvement of cracks by a single auxiliary field is limited. Therefore, some scholars conduct research in the compound field. First, find the inflection point of the residual stress through a single factor, and then find the optimal parameter combination of the composite field through the comparison method, the orthogonal test method, or the response surface method. Zhang et al. [96] introduced a hybrid ultrasonic–electromagnetic field and concluded through the comparison method that the composite electromagnetic field can alleviate temperature gradients and melt pool inhomogeneity. This approach promoted the transformation of coarse, blocky carbides into a particulate morphology, thus minimizing crack formation. Simultaneously, the sedimentation of WC particles towards the coating’s base was suppressed, leading to a more uniform distribution of WC particles throughout the coating.

In summary, friction stirring typically relies on thermal coupling, electromagnetic fields use Lorentzian magnetism for stirring, and ultrasonic vibration utilizes the cavitation effect and the acoustic flow effect. All of these measures inhibit cracking. While individual auxiliary measures can effectively mitigate defects within the cladding, combining them yields superior enhancement of coating performance. Both single-assisted and composite-assisted techniques reduce the susceptibility to cracking by reducing the accumulation of stresses and refining the grain structure. Consequently, the implementation of auxiliary measures during the cladding process is of significant importance in enhancing the homogeneity of the coating microstructure, refining the grain size, reducing residual stresses within the coating, and mitigating the formation of cracks and porosity defects.

#### 4.3.3. Improvement of Cracks by Heat Treatment

Heat treatment is an important process for improving the performance of coatings. According to the time of action, it can be classified into preheating treatment of the substrate before cladding, post-heat treatment after cladding completion, and laser remelting. Preheating before forming can effectively reduce the non-uniformity of the temperature field and reduce the mutual accumulation of stress between different welding passes [97]. Post-heat treatment and laser remelting at the completion of cladding can reduce the yield strength of the material in the stress area, promote the release of stress, and thereby reduce the occurrence of cracks.

Preheating involves heating the substrate as a whole or locally to a certain temperature and using a laser to prepare the cladding coating on the hot substrate. This process can reduce the temperature gradient, increase the duration of the molten pool, homogenize the temperature field, and prevent cracks caused by phase change. However, excessive preheating will greatly increase the height of the cladding layer, preventing the adequate expulsion of gases and slag from the lower layers, leading to defects. Therefore, selecting the appropriate preheating temperature is a key factor in improving the performance of the coating. Wu et al. [98] used 60Si2Mn steel as the matrix to study the effects of the morphology, the microstructure, and the wear resistance of cobalt-based tungsten carbide composite coatings at preheating temperatures of 150 °C, 250 °C, and 350 °C, respectively. The results show that the coating height increases from 0.49 mm to 2.41 mm, and the cladding angle decreases sharply with the increase in the preheating temperature. When the substrate is preheated to 350 °C, the coating exhibits optimal hardness and wear resistance. On this basis, Zhang Guangtai et al. [99] made a comparison between preheating of the substrate and normal temperature, and they demonstrated that substrate preheating reduced the internal stress induced via non-uniform solidification rates across different regions of the coating, thereby effectively preventing crack formation.

Laser remelting builds upon laser cladding by employing a secondary laser scan across the cladded, which helps mitigate residual stresses within the coating. Zhou et al. [100] fabricated a WC-reinforced iron-based amorphous composite coating. The study demonstrated that cracks and pores were eliminated and the surface became smooth after laser remelting treatment, and the corrosion resistance and hardness of the coating were superior to those of the non-remelted laser cladding coating. Lv et al. [101] applied laser remelting to FeCoNiCrAl high-entropy alloy coatings, and observed that the atomic proportion of Al element on the surface of the coatings increased from 12.56% to 20.31%, The structure of the coatings transformed from FCC, A2, and B2 phases to a single BCC structure, and the grain morphology changed from elongated plate-like grains to equiaxed grains. As a result, surface defects in the coating were effectively repaired, leading to significant improvements in surface hardness and wear resistance.

Heat treatment involves heating a metal material to a specific temperature, retaining it for a period of time, and then cooling it in an appropriate manner to improve the material’s properties. This process enhances the ductility and toughness of alloy materials while also mitigating residual stresses and hardening effects, thereby facilitating subsequent machining and forming operations. Common heat treatment methods include normalizing, annealing, and solution treatment, all of which can enhance coating performance. These techniques are all capable of improving coating performance. Du et al. [102] applied Fe60 coatings on 304 stainless steel substrates, followed by normalizing and solution heat treatments. The results indicated that these treatments reduced the internal energy of the coating, transforming the columnar or cellular grains into equiaxed grains and refining the microstructure, and reduced lattice distortion. The increased number of grains enhanced the dispersible plastic deformation, thereby effectively reducing crack formation. Although both normalizing and solution treatments led to a decrease in microhardness, the tensile properties and impact toughness were significantly improved. Dai et al. [83] reduced coating cracks by using a combination of substrate preheating and annealing. Chen et al. [103] employed laser cladding to deposit Stellite 6 alloy onto the surface of 1Cr11Ni2W2MoV steel. Their investigations revealed that cracks initiated in the coating when the thickness exceeded 3 mm. Subsequent annealing at 650 °C for 2 h mitigated stress concentration, resulting in the elimination of cracks and an enhancement in the coating’s erosion resistance.

Through the summary of the three heat treatments, this study confirms that preheating the substrate is the best way to improve cracks; it directly reduces the thermal stress in the coating. Although laser remelting releases residual stress, it introduces new stress. Post-heat treatment cannot completely eliminate the existing cracks. Furthermore, when a single heat treatment method cannot completely eliminate coating defects, the combined use of heat treatment will become a new approach to improving cracks.

#### 4.3.4. Auxiliary Software for Crack Prevention and Control Measures

In experimental studies, it is challenging to continuously observe the entire laser cladding process, including powder melting, molten pool formation, internal convection, solidification, and cladding layer development. Therefore, numerical simulation has become an effective method for understanding the cladding process. By establishing a multi-physical field coupling model of heat, flow, and stress, researchers can not only visualize complex physical phenomena but also predict crack propagation, molten pool morphology, and stress distribution, thereby providing a theoretical basis for optimizing process parameters. However, in the process of developing predictive models that can adapt to multiple material systems and complex process settings, many challenges are still faced.

Firstly, different alloy systems (such as Co, Ni, and Fe-based) vary greatly in physical properties such as thermal conductivity, specific heat, and the melting point, which limits the applicability of the general model. Secondly, there is a complex nonlinear coupling among laser power, scanning speed, powder composition, and auxiliary fields (such as ultrasonic or magnetic fields), which enhances the model’s sensitivity to parameter changes. In addition, the limited experimental data, the difficulty in accurately setting the boundary conditions, and the combined effect of multiple factors such as phase transition, pores, and cracks make the high-precision prediction of the model difficult.

**Figure 9:**
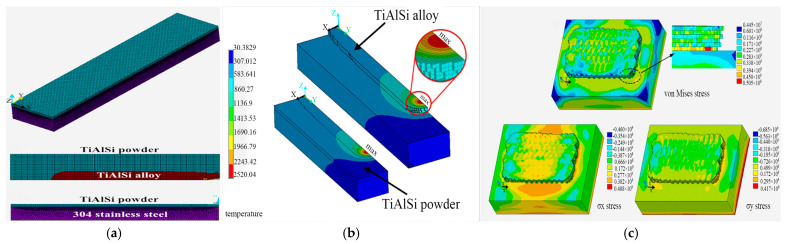
(**a**) Finite element model. (**b**) Distribution of molten pool temperature [104]. (**c**) Distribution of residual stress [105].

To deal with the above problems, researchers have adopted a variety of strategies. Shen et al. [104] simulated the temperature evolution of the molten pool and the geometric morphology of the cladding layer during the laser cladding process of TiAlSi coating through an improved three-dimensional transient finite element model (Figure 9a,b), predicted the geometric dimensions of the coating under different process parameters (power and speed), and verified the accuracy of the simulation results through experiments. Li et al. [105] and others developed a three-dimensional thermal–structural coupling model a three-dimensional thermal–structural coupling model to simulate the multilayer multi-channel SLM process, and they analyzed the temperature field, melt pool size, stress field, and residual stress distribution. The results show that the melt pool size increases with the increase in temperature, and the error between the simulated melt pool size and the experimental results is within 10%. The stress distribution exhibits compressive stress in the high-temperature region, while the low-temperature region exhibits tensile stress during the cooling process. The maximum residual stress occurs at the connection between the first pass of the first layer and the substrate (Figure 9c). Ye et al. [106] established a multiphysics model based on Comsol 5.6 software and revealed the regulatory mechanism of Marangoni convection on the uniformity of the molten pool flow field. Ma et al. [107] investigated the influence of key process parameters on coating quality during laser cladding. A polynomial regression model was developed using RSM to describe the effects of various factors on dilution rate and residual stress. To further optimize these parameters, a multi-objective quantum-behavior particle swarm optimization (MOQPSO) algorithm was employed. The results demonstrated that MOQPSO effectively optimized both the dilution rate and residual stress, and a set of Pareto-optimal solutions was successfully obtained. Tian et al. [108] constructed a 3D thermodynamic finite element model to investigate stress-induced solid-state phase transformation (SSPT) effects on residual stress evolution during cladding. The simulations revealed that SSPT drives stress-assisted austenite-to-martensite conversion, reducing lateral and longitudinal residual stresses.

Let us sum up. In recent years, prediction models have been evolving from a single physical field to multi-field coupling. The optimization of parameters in the simulation has also shifted to intelligent algorithms. In the future, the generalization ability and prediction accuracy of the model under cross-material and variable process conditions can be further enhanced by introducing material characteristic parameters, constructing a structured process–performance database, and adopting machine learning algorithms to assist in modeling.

#### 4.3.5. Improvement of Coatings Through Other Means

In order to produce defect-free and high-performance fused coatings, researchers are exploring new preparation processes and new structural materials.

In new preparation processes, Yang et al. [109] fabricated high-reflectivity CuCrZr alloy-clad coatings on AlSi7Mg substrates, systematically investigating deposition quality and solidification characteristics under red, blue, and hybrid laser conditions. The results indicated that problems such as cracking and spheroidization occurred in the coatings when a high-power infrared laser (2600 W) and a blue light laser (960 W) were used. When the high-power infrared and blue light lasers were used in combination, macroscopic segregation and Al2Cu precipitation occurred in the fusion coating, and the coating remained cracked. When using a combination of blue light and low-power red light, no segregation or cracking was observed in the coating. The hybrid laser approach offers a new method for preparing high-quality fused coatings. In addition, after research by scientists, subsequent research demonstrated that the hybrid process also significantly improved the quality of the coatings. Compared with the traditional laser cladding, the composite process can effectively reduce the defects of the coating, significantly improve the performance of the coating, and also reduce the number of experiments and subsequent material processing. Zhang et al. [110] combined laser fusion deposition with shot peening to enhance surface quality and mechanical performance. High-speed shot peening was used to remove the surface oxide layer and smooth the joint surface, thereby improving surface roughness and dimensional accuracy. In addition, shot peening transformed the residual tensile stress in the specimens into compressive stress, effectively mitigating issues such as porosity associated with high residual tensile stress. In addition to the hybrid process, the laser system, scanning system, powder delivery system, process parameters, thermal management, intelligent control, and other aspects are optimized and improved on the basis of the traditional laser cladding to form a new process called ultra-high-speed laser cladding. Ultra-high-speed laser cladding (UHSLC) is characterized by high powder utilization, thin coating thickness, and a low heat input. Ding et al. [111] applied Inconel 625 coating on 27 SiMn substrate using ultra-high-speed laser cladding technology and traditional laser cladding technology, respectively. The results showed that UHSLC exhibited higher cladding efficiency, a lower heat input, and better coating performance when Inconel 625 coatings were prepared. In terms of performance, the ultra-high-speed laser cladding technology transformed the coarse columnar crystals in the coating into fine dendrites, and the hardness, wear resistance, and corrosion resistance of the coating are improved. In addition, UHSLC technology also reduces surface roughness, making the solid coating surface smoother.

In terms of new structural materials, Liu et al. [112] and others combined the advantages of eutectic and high-entropy alloys to develop a novel Al27Cr25Ti18Nb18Zr12 eutectic high-entropy alloy coating. The coating features a nano-layered eutectic structure, comprising BCC, B2, and laves phases. A large number of dislocation locks in the BCC and B2 phases impede material deformation, enhancing hardness and preventing adhesion during wear. This structure effectively addresses the adhesion issue commonly encountered with titanium alloys. Li et al. [113] and others employed laser cladding technology to synthesize core–shell-structured ceramic particles in situ within an iron-based composite coating. The core–shell ceramic particles consisted of a pure TiC core and a (Ti, Nb, W)C composite carbide shell, with microparticles (0.5~1.0 μm) uniformly distributed in the fusion-coated layer. This uniform distribution of core–shell particles significantly improves the microstructure of the coating, resulting in a more uniform hardness distribution, reduced susceptibility to cracking, and a tenfold increase in wear resistance relative to the original. The design of this core–shell structure also solves the problems of gradient distribution and crack formation that exist in traditional ceramic particle-reinforced coatings.

In summary, researchers have explored new processes and new structural materials that have led to tremendous improvements in coating performance. These methods provide new solutions for the preparation of high hardness, high wear resistance, high corrosion resistance, and crack-free coatings.

## 5. Summary and Outlook

This article has reviewed the laser cladding material system, process parameters, crack formation mechanism, and control methods. In laser cladding, the material system is primarily categorized into iron-based autosoluble alloy powder, cobalt-based autosoluble alloy powder, nickel-based auto-soluble alloy powder, and ceramic powder. The main process parameters are laser power, the spot diameter, the scanning speed, the powder feeding method, and protective gas. The selection of a material system significantly influences the thermal stress distribution, while the optimization of process parameters can further regulate the temperature field. Therefore, in the selection of materials, good wettability and similar physical properties, such as the coefficient of thermal expansion and thermal conductivity, should be considered between the cladding material and the substrate. In terms of process parameter optimization, algorithms can be employed to determine the optimal solution, enabling both single-objective and multi-objective optimization of process parameters to achieve high-quality coatings with low dilution, favorable morphology, and superior hardness and wear resistance.

Cracking is a significant challenge in laser cladding. Cracking is primarily caused by internal and residual stresses. Due to the differences in the coefficient of thermal expansion and modulus of elasticity between the cladding material and the base material, the uneven distribution of stress and temperature fields in the melt pool can lead to the accumulation of internal and residual stresses, resulting in cracks. In addition, defects such as impurities and porosity in the coating also increase the sensitivity to cracking. Therefore, current research focuses on regulating internal stresses and minimizing stress concentration to inhibit or eliminate cracking during laser melting and cladding.

Regarding the preventive measures for cracks, this article systematically summarizes them into five key aspects: process selection during the preparation phase, the application of auxiliary methods in the cladding process, heat treatment techniques, the utilization of specialized software, and the exploration of new processes and advanced structural materials.

(1) During the selection of processes in the preparation stage, the temperature gradient can be effectively mitigated by selecting materials with similar coefficients of thermal expansion, incorporating materials with negative expansion coefficients, rare earth elements, nanoparticles, and alloying components. Additionally, the optimization of process parameters can significantly enhance the uniformity of the temperature field, mitigate stress accumulation between adjacent cladding weld beads, and consequently minimize the incidence of crack formation.
(2) The application of auxiliary scenarios in the cladding process, through the use of friction stirring, ultrasonic vibration, electromagnetic fields, and composite fields, can homogenize the melt pool and refine the grain structure, thereby reducing stress concentration and crack sensitivity.
(3) Heat treatment technology, through the preheating treatment of the substrate, post-heat treatment after cladding completion, and the application of laser remelting technology, improves the defects of the coating.
(4) Auxiliary software can be used to construct models that analyze the temperature and stress fields in the melt pool during the cladding process and to predict the formation of cracks. This not only helps reduce the cost of experiments but also provides a new way to prepare high-quality coatings by laser cladding.
(5) New preparation processes and new structural materials. These include the use of hybrid laser sources, laser cladding composite processes, ultra-high-speed laser cladding technology, nanolayered eutectic organization, and ceramic particles with a core–shell structure. All of these techniques improve the coating properties, resulting in a huge increase in hardness, wear resistance, and corrosion resistance.

Through the above measures, the microstructure of the coating is improved, while typical defects such as pores and cracks are reduced, and the durability of the material under cyclic loading and service environments is also enhanced.

In addition, with the continuous increase in the demand for material performance in high-end service environments, laser cladding technology has shown great application potential in multifunctional composite coatings. By rationally designing the alloy system and the synergistic combination of reinforcing phases, such as introducing wear-resistant ceramic particles (such as WC and TiC), corrosion-resistant elements (such as Cr and Mo), and high-temperature resistant phases (such as Al_2_O_3_ and Y_2_O_3_), and combinations with process optimization and defect prevention measures, the synergistic enhancement of multiple properties such as wear resistance, corrosion resistance, and thermal stability can be achieved. Without significantly sacrificing the toughness and bonding strength of the coating, laser cladding has the ability to construct functional gradient or composite structures that integrate “multiple effects in one layer”, providing customized protection for equipment components under extreme working conditions (such as high temperature, high load, and strong corrosion). Therefore, in order to obtain high-quality coatings, the following aspects can be considered in the future.

(1) With regard to rare earth elements and hard particles, different hard particles have different physical and chemical properties, and synergistic use of a variety of hard substances can be considered to meet the needs of multiple applications. The addition of rare earth elements can improve coating performance, but the cost is too high. Consideration should be given to developing new composite materials to reduce costs.
(2) In terms of new processes, it is possible to consider combining other surface treatment processes with laser cladding to take advantage of their own advantages and further improve the optimization of the coating.
(3) In terms of process parameters, the optimization of algorithms to find the optimal process parameters, or the improvement of equipment (e.g., nozzle angle, different laser source, etc.) may be effective.
(4) In terms of the auxiliary simulation, the simulation of the auxiliary software sometimes exhibits partial differences with the actual experimental data. Therefore, the distribution of the temperature field and the stress field in the simulation is improved in the following research to provide authenticity for the experiment.
(5) In terms of automation, laser cladding can be combined with automation to improve the automation system and real-time monitoring and feedback. As a result, the melting efficiency, coating quality, safety, and cost reduction can be improved.

## Figures and Tables

**Table 1:** Optimization of process parameters.

Method	Optimization Goal	Optimized Parameters	Performance of Coating	Reference
Orthogonal experimental design method and GABP-NSGAII multi-objective optimization algorithm	B4C content, P and V	B4C content was 15 wt.%, P: 2200 W, V: 800 mm/min	The microhardness of the coating is 434 HV0.5,which is increased by 33.16%.	[55]
Response surface method	P, V, Vf, η, W/H and θ	P: 1477 W, Vf: 17.5 mg/s, V: 5 mm/s, η: 0.079, θ: 145°W/H: 5.13	Without cracks, deformation and pores, the microhardness of the coating (620 HV0.2) is 3.1 times that of the 45 steel matrix.	[56]
Gray correlation analysis and variance analysis	P, V, Vf	P: 2100 W, V: 6 mm/s, Vf: 17.90 g/min	Without cracks, holes and element segregation, the hardness, toughness and corrosion resistance of the coating are significantly improved.	[57]
Generalized Regression Neural Network Algorithm (GRNN) and Non-Dominated Sorting Genetic Algorithm (NSGA-Ⅱ)	P, V, Vf	P: 2.33 KW, Vf: 12.46 g/min, V: 2.9 m/min, preheating temperature 400 °C	Without cracks, the microhardness and wear resistance of the coating are significantly improved.	[58]
Random Forest Regression using the Osprey Optimization Algorithm (OOA-RFR) and Unified Non-dominated Sorting Genetic Algorithm III (U-NSGA-III)	P, V, Vf, Φ, and Ti, B_4_C mixed powder content	P: 1587 W, V: 11 mm/s, Vf: 1.3 r/min, Φ: 50%, the content of mixed powder is 11%	The surface smoothness, microhardness and wear resistance of the coating are significantly improved.	[59]
Orthogonal experimental design, Particle Swarm Optimization (PSO), Genetic Algorithm (GA) and Non-dominated Sorting Genetic Algorithm II (NSGA-II)	P, V, Vf	P: 934 W, V: 352 mm/min, Vf: 0.64 r/min, W/H: 3.06, θ: 0.33	Without defects, the hardness of the coating is 613 HV, which is three times that of the substrate.	[60]
Response surface design method	P, V, Vf	P: 431 W, V: 5.34 mm/s, Vf: 1.03 r/min	The microstructure was refined and the microhardness of the coating was increased to 470.8 HV.	[61]

**Table 2:** The influence of alloying elements on coating properties.

Coating/Substrate	Alloy Content wt.%	Improvement Effect
WC-Ni60AA/65Mn [80]	0.25%/0.50%/0.75%/1.0%/1.25% Mo	Strengthening of grain boundaries, refinement of tissue, disappearance of cracks at 1.0% Mo content.
Ni60A-WC/45 steel [81]	2%/5%/10%/15%/20% Ta	Reduces WC size and promotes uniform hard phase distribution, with minimal cracking at 10 wt.% Ta powder addition.
Inconel625/Q235 steel [82]	1.5%/3%/5% Nb	The transformation of the coating organization from dendritic to equiaxial crystals and the generation of a passivation film on the surface resulted in the best hardness and corrosion resistance of the coating at 3% Nb.
Ni50A-WC/H13 steel [78]	3%/6%/9% Ti	Refining the carbides and borides of Cr improves melt pool fluidity and reduces the number of cracks due to porosity, with cracks and porosity disappearing at 3% Ti.
Fe-WC/0.45% medium carbon steel (AISI 1045) [83]	8% Co	Improve the toughness of the coating, reduce brittle fracture and lower crack sensitivity.
CoCrMo/24CrNiMo Cast steel [84]	13% Ni, 7% Al	The microstructure of the coating was transformed from a network structure to a sawtooth structure, increasing the ductility of the coating by 53% and reducing the rate of thermal fatigue crack propagation by 40%.

## Data Availability

No new data were created or analyzed in this study. Data sharing is not applicable to this article.
